# Salivary Testosterone, Androstenedione and 11‐Oxygenated 19‐Carbon Concentrations Differ by Age and Sex in Children

**DOI:** 10.1111/cen.15258

**Published:** 2025-05-20

**Authors:** Julie Park, Andrew Titman, Orla Bright, Silothabo Dliso, Alena Shantsila, Gregory Y. H. Lip, Jo Adaway, Brian Keevil, Daniel B. Hawcutt, Joanne Blair

**Affiliations:** ^1^ Department of Endocrinology Alder Hey Children's NHS Foundation trust Liverpool UK; ^2^ Department of Women's and Children's Health University of Liverpool Liverpool UK; ^3^ Liverpool Centre for Cardiovascular Science at University of Liverpool, Liverpool John Moores University and Liverpool Heart & Chest Hospital Liverpool UK; ^4^ School of Mathematical Sciences Lancaster University Lancaster UK; ^5^ NIHR Alder Hey Clinical Research Facility Alder Hey Children's Hospital Liverpool UK; ^6^ Department of Clinical Medicine, Danish Center for Health Services Research Aalborg University Aalborg Denmark; ^7^ Clinical Biochemistry Department University Hospital of South Manchester Manchester UK

**Keywords:** 11‐oxygenated 19‐carbon steroids, adrenal, androgen, biomarker, paediatric, saliva, steroid

## Abstract

**Background:**

The diagnosis and management of childhood adrenal disorders is challenging. Clinical markers of hormone excess or deficiency may take months to manifest, and traditional biomarkers correlate only partially with clinical outcomes. Recent work has indicated that 11 oxygenated 19‐carbon (11oxC19) steroids may be useful in the assessment of adrenal function. 11oxC19 steroids, testosterone (T) and androstenedione (A4), can be measured in saliva, but very little is known about these hormones in healthy children.

**Methods:**

Participants collected saliva samples 30 min after waking and every 2 h until bedtime. Samples were analysed for T, A4, 11 ketotestosterone (11KT) and 11βhydroxyandrostenedione (11OHA4) by liquid chromatography tandem mass spectrometry.

**Results:**

Fifty‐two (30 male) healthy children aged 10.4 ± 3.9 (5.0–17.5) participated. Median height SDS was 0.4 (IQR −0.3 to 1.01) and median BMI SDS was 0.3 (IQR −0.2 to 1.3). All steroids showed a diurnal rhythm, with all hormones decreasing in measured concentration at time points that are 30 min after waking. Salivary T was higher in postpubertal children, particularly boys (*p* < 0.001). Salivary A4 was lower in boys compared to girls (*p* = 0.009) and did not differ with pubertal development. 11KT increased with age (*p* < 0.001) and concentrations were similar between boys and girls. 11OHA4 reduced in concentration with age (*p* = 0.03) and was below detectable limits after the early morning peak in both sexes.

**Conclusion:**

For the first time we describe the physiological profile of 11KT and 11OHA4 in children. Further data are required to establish reference ranges, which should consider age, sex, pubertal status and time of sampling.

## 1 Introduction

The diagnosis and management of children with adrenal and gonadal disorders is challenging. Symptoms of hormone excess or deficiency may be vague and non‐specific while clinical signs may take months to become evident, and the diurnal profile of hormones released from the adrenal can only be documented by collecting multiple samples over the course of 24 h.

Disorders of adrenal disease, such as congenital adrenal hyperplasia (CAH) can be monitored using biochemical markers such as androstenedione (A4), testosterone (T) and 17hydroxyprogesterone (17OHP). Emerging evidence suggests that 11‐oxygenated 19‐carbon (11oxC19) steroids can also be used. Little are known about normative salivary concentrations in healthy children.

11oxC19 are adrenal derived steroids and include 11 ketotestosterone (11KT), 11β‐hydroxy testosterone (11OHT), 11‐ketoandrostenedione (11KA4) and 11β‐hydroxyandrostenedione (11OHA4). 11oxC19 steroids are synthesized from A4 and testosterone by the cytochrome P450 11β‐hydroxylase (CYP11β1) which is found predominantly in the adrenal cortex. They have little or no gonadal secretion compared to other more traditional biomarkers, such as A4 or T [1]. Salivary and serum 17OHP, A4, T, 11KT and 11OHA4 correlate well in children with CAH [2].

There is emerging evidence that 11‐oxygenated 19‐carbon (11oxC19) steroids are raised in disorders of androgen excess, including CAH [3, 4], Cushing's disease [5] and adrenal adenomas [6] and may be more informative than T and A4. Higher concentrations may be associated with testicular adrenal rest tumours (TART) [7] and larger volume adrenal glands on CT [7] in patients with CAH, and therefore poorer outcomes.

A recent study reported diurnal profiles of 17OHP and A4 in saliva in adults and young children, and pre‐pubertal and adult reference ranges have been reported. Boys aged 11–15 years and girls aged 10–15 years were excluded from this study as pubertal status was not recorded. The peri‐pubertal cohort has also been excluded from previous studies [8].

In this study, we report salivary T, A4, 11KT and 11OHA4 concentrations in healthy children, in samples collected 30 min after waking and 2‐hourly throughout the day. We also explore relationships between these measurements, age, and sex.

## 2 Materials and Methods

The study was approved by Cornwall and Plymouth Research Ethics committee (REC Ref: 20/SW/0162). Consent and assent were obtained in line with Good Clinical Practice guidance.

### 2.1 Participants

The following individuals were invited to participate:

- Healthy siblings of patients attending our hospital.
- Children attending our hospital for treatment of a conditions not associated with an abnormality of adrenal function.
- Children of staff working at our hospital or the University of Liverpool.

Information about the study was distributed by hospital communications team, and with parents during hospital appointments. Children aged 5–18 years were eligible to participate, and those with oral conditions likely to result in blood contamination of saliva samples and those with a family history of adrenal insufficiency due to an inherited condition were excluded. Study participants were recruited between December 2021 and April 2022.

### 2.2 Study Procedures

Participants attended the NIHR Alder Hey clinical research facility (CRF) where written, informed consent and assent were obtained. Demographic data were collected including age and sex. Height and body mass index (BMI) standard deviation scores (SDS) were derived from reference data according to age and sex [9]. Tanner staging of puberty was not performed, as it was felt that this was likely to be a significant deterrent to participation. Pre‐puberty was defined as age less than 9 years in females and less than 10 years in males.

Participants were trained to collect saliva samples using Salivettes (*Salimetrics, New Market*). Samples were collected 30 min after waking, and then 2‐hourly until bedtime. Children were asked not to eat, drink or brush their teeth an hour before obtaining the salivary sample. Samples were stored at home in a domestic freezer until return to the CRF. Saliva samples were then stored at −80° centigrade until analysis.

### 2.3 Sample Analysis

Saliva (250 µL) was mixed with isotopically labelled internal standards and extracted by supported liquid extraction using 1 mL of methyl tert‐butyl ether. Chromatography was performed using a Waters Acquity HSS T3 (1.8 µm, 100 Å, 2.1 × 50 mm) column using a gradient of 2 mmol/L ammonium acetate and 0.1% (v/v) formic acid in water and acetonitrile, and quantification was performed on a Waters TQ‐XS mass spectrometer. The lower limit of quantitation was 5 pmol/L for T, 10 pmol/L for A4, 12.5 pmol/L for 17OHP, 6 pmol/L for 11KT, and 45 pmol/L for 11OHA4. The assay was linear up to 50,000 pmol/L for T, 100,000 pmol/L for A4, 150,000 pmol/L for 17OHP, 120,000 pmol/L for 11KT, and 250,000 pmol/L for 11OHA4. Recovery was shown to be 92.5%–109.8% for all analytes, inter‐assay precision was 92.5%–109.8% for all analytes, inter‐assay precision was < 8.6% (< 13.7% at lower limit of quantitation (LLOQ) and inter‐assay bias from nominal concentrations was < 8.8% (< 15.6% at LLOQ).

### 2.4 Statistical Analysis

To account for within and between patient variability in the measurements across time, linear or non‐linear mixed effects models [10] are fitted to each of the biomarkers. Specifically, the linear mixed effects models assume

$$\log\left(y_{\mathrm{ij}}\right)=\left(\alpha +a_{i}\right)+\left(\beta +b_{i}\right)t_{\mathrm{ij}}+\gamma^{T}z_{i}+\epsilon_{\mathrm{ij}},$$

where $\epsilon_{\mathrm{ij}}\sim N(0,\sigma^{2})$ are mutually independent residuals, and ${\left(a_{i},b_{i}\right)}^{T}$ are patient‐specific random intercepts and slopes which follow a bivariate normal distribution with mean zero and variance‐covariance matrix. Here **z_i** is a vector of patient specific covariates, principally sex and age or sex and assumed pubertal status. Interactions between sex and age were also considered. The presence of a diurnal trend can be tested by assessing whether β is significantly different from zero.

Exploratory analysis indicated that for all biomarkers except 11‐ketotestosterone, a linear trend on the log‐scale was not appropriate. Instead, the values tended to initially decrease rapidly before plateauing. To accommodate this possible trend a non‐linear mixed effect model was considered of the form:

$$\log\left(y_{\mathrm{ij}}\right)=g\left(t_{\mathrm{ij}};\theta ,\;u_{i}\right)+{\gamma^{T}z_{i}+\epsilon }_{\mathrm{ij}},$$

where

$$g\left(t;\theta ,\;u\right)=\theta_{1}+u_{1}+\left(\theta_{2}+u_{2}\right)\text{exp}\left(-\theta_{3}t\right).$$

Here $\left(u_{1},u_{2}\right)$ are patient‐level random effects with a bivariate normal distribution. Here $\theta_{1}$ determines the ultimate (e.g,. end‐of‐day) plateau level of the biomarker, $\theta_{2}$ represents the amount by which the log‐biomarker value decreases to its plateau, and $\theta_{3}$ defines the suitability of a log‐transformation was assessed by considering the best fit of a linear model under the family of Box‐Cox transformations [11].

Both the linear and non‐linear mixed effects models were fitted using maximum likelihood estimation to allow model comparisons through AIC and likelihood ratio tests.

For 11OHA4 a substantial proportion of all observations are below the limit of detection. This is a form of left censoring. To account for this in the model fitting, censored regression techniques are used where the likelihood of observations below the limit of detection, ld, are taken to be $P\left(\log\left(y_{\mathrm{ij}}\right)<\mathrm{ld}\right)$ [10]. Specifically, a linear mixed effects model with a random intercept is considered.

The absolute goodness‐of‐fit of models is assessed by comparing the trend in the median with the sample mean at each time point. Pointwise 95% confidence intervals for the sample mean are also presented. These are computed for the model that does not account for the effect of sex and age. To account for left‐censoring induced by limits of detection in 11OHA4, a normal distribution with unknown mean and variance is fitted to the data from each time point, independently (treating values below ld as left‐censored). The maximum likelihood estimate of the mean parameter is then taken as the estimate.

All statistical analyses were carried out using R version 4.3.1. The standard linear mixed effects models and non‐linear mixed effects models are fitted using the lme4 package (version 1.1‐34). The censored linear mixed effect models are fitted using the censReg package [(version 0.5‐38) [12].

## 3 Results

The study recruited 52 (30 male) participants aged 10.4 ± 3.9 (5.0–17.5) years. Median BMI SDS was 0.3 (IQR −0.2 to 1.3) and median height SDS was 0.4 (IQR −0.3 to 1.01). All participants were born between 37 and 42 weeks with a median BW SDS ‐0.1 (range −2.3 to 2.2). 74.1% of participants were white British, 12.1% were Asian and 7.4% white European. Deprivation score calculated by IMD decile determined that 9 (16.7%) were in class 1–2, 8 (14.8%) were in 3–4, 12 (22.2%) were in 5–6, 14 (25.9%) were in 7–8 and 11 (20.4%) were in 9–10, with 1 being the most deprived area and 10 being the least deprived. Blood pressure (BP) was normal (< 90th centile) in 83.3% of participants. In five children diastolic or systolic BP was between 90 and 95th centile and four above the 95th centile for age and height.

For all biomarkers except 11OHA4, a Box‐Cox transformation analysis on a linear model involving an interaction between age and sex indicated that the optimal transformation was very close to a log‐transformation. For 11OHA4, this was only the case if the Box‐Cox transformation was applied to the model accounting for left‐censoring.

### 3.1 Androgens Synthesised by the Classical Pathway

#### 3.1.1 Testosterone

**Figure 1:**
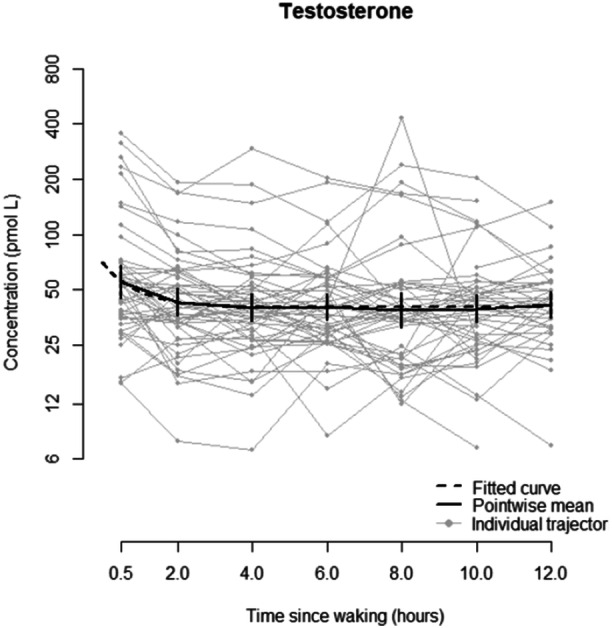
Concentration of testosterone compared to time since waking (hours), showing the fitted curve, pointwise mean and individual trajectories.

In total, 276 samples were available from 54 participants (30M) and the volume of saliva was insufficient in 12 samples. For testosterone, there is evidence of the value plateauing after an initial drop in the first few hours after waking (Figure 1). Therefore, a non‐linear mixed effects model fitted the data considerably better than a linear mixed effects model (AIC 413.5 vs. 427.0). There was strong evidence for a diurnal pattern (*p* = 0.005), where the estimated mean decrease, $\theta_{2}$ 0.434 (95% CI: 0.087, 0.782). Full details can be found in Appendix 1. A model accounting for age by assuming a linear effect fitted substantially better than using assumed pubertal status (AIC 413.5 vs*.* 429.0).

Only a model with a random value for $\theta_{1}$ (controlling the intercept) converged for this data set. There is evidence of a strong effect of sex and a substantial interaction with age, where males have generally higher concentrations of testosterone and their concentration increases with age, whereas female's testosterone levels do not have a discernible trend with age. A male of age 10.3 years (the sample average) is estimated to have a testosterone level 30.3% (95% CI: 9.6%, 43.5%) higher than the same aged female. Male's testosterone level is estimated to increase by 12.6% (95% CI: 9.3%, 16.0%) per year of age, whereas there is no significant trend in age for females (0.2% per year, 95% CI: −3.3%, +3.7%).

#### 3.1.2 Androstenedione

**Figure 2:**
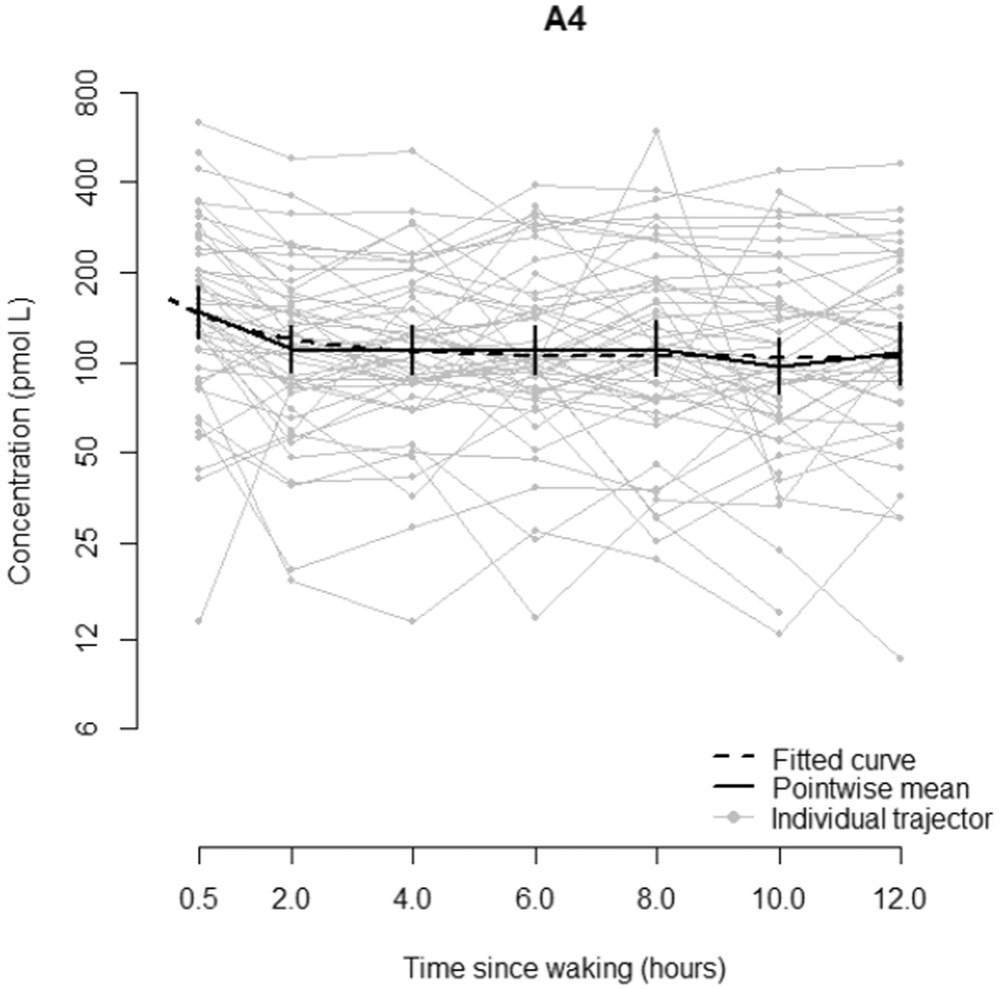
Concentration of androstenedione (pmol/L) compared to time since waking (hours), showing the fitted curve, pointwise mean and individual trajectories.

In total, 281 samples were available for analysis, and saliva volume was insufficient in seven samples. For A4, there is an initial reduction in concentration between 0.5 and 2 h after waking and then an apparent plateauing (Figure 2). As such, a Non‐linear mixed effects model is most appropriate, although the improvement in fit compared to a linear mixed effects model is not substantial (AIC 485.0 vs. 487.3). The decreasing trend in time is statistically significant *p* = 0.009). There is no significant effect of age or interaction between age and sex (this is also the case if assumed pubertal status is used rather than age). Full details can be found in Appendix 1.

### 3.2 11‐Oxysteroid Pathway Metabolites

#### 3.2.1 11‐KT

In total, 314 samples were available for analysis and 28 samples were insufficient for analysis. 11KT was undetectable in 19 (6.1%) samples. Samples in which 11‐KT concentrations were below detectable levels (< 6 pmol/L) were recorded as 5 pmol/L for statistical analysis.

**Figure 3:**
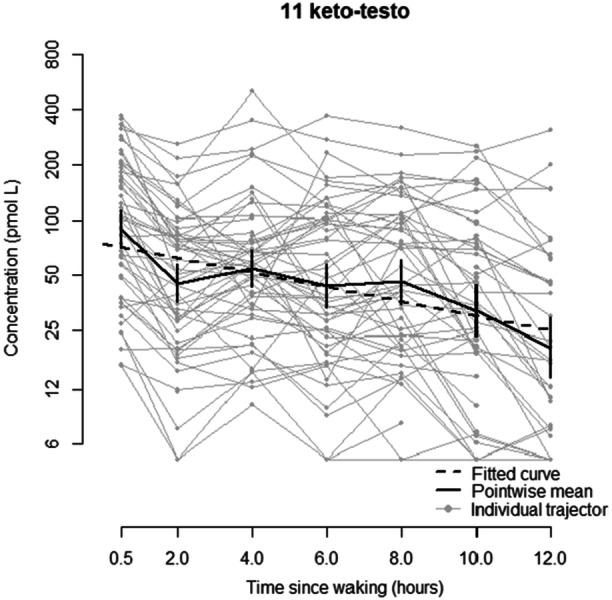
Concentration of 11 ketotestosterone (pmol/L) compared to time since waking (hours), showing the fitted curve, pointwise mean and individual trajectories.

For 11 keto‐testosterone, a linear mixed effects model with a random intercept and slope provides a good fit to the data. There is a statistically significant decreasing trend in log levels with time since waking (*p *< 0.0001), with levels decreasing on average by 8.8% (95% CI: 7.4%, 10.2%) per hour since waking (Figure 3). 11 keto‐testosterone levels increase with age for both males (increase of 21.0% per year of age, 95% CI: 15.6%, 26.8%) and females (increase of 14.3% per year of age, 95% CI: 8.1%, 20.8%), although the difference in trends between males and females is not statistically significant. As with testosterone, a model assuming a linear effect of age fits substantially better than one using assumed pubertal status as a binary covariate (AIC 625.4 vs. 639.4). Full details can be found in Appendix 1.

#### 3.2.2 11OHA4

**Figure 4:**
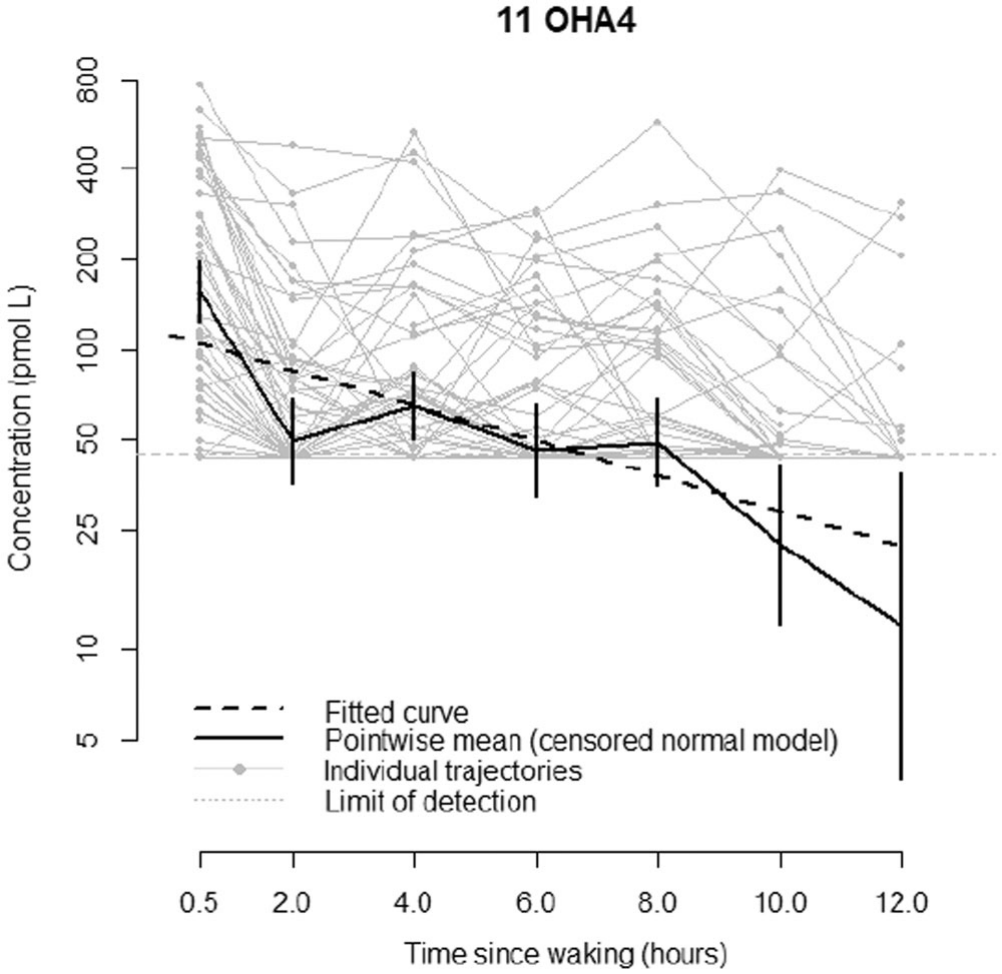
Concentration of 11B‐hydroxyandrostenedione (pmol/L) compared to time since waking (hours), showing the fitted curve, pointwise mean and individual trajectories.

In total, 325 samples were available for analysis, and hormone concentrations were undetectable (< 45 pmol/L) in 148 samples. For 11OHA4 a high proportion of all observations are below the limit of detection (45 pmol/L). Rather than naively imputing a value for these values, a linear mixed effects model assuming values below the limit of detection are left‐censored at 45 pmol/L, can be fitted (Figure 4).

There is statistically significant decrease in log‐concentration with time since waking (*p* < 0.0001), with levels estimated to decrease on average by 12.6% per hour since waking (95% CI: 10.2%, 14.9%). There is also a statistically significant decrease in concentration with age (*p* = 0.03) with an estimated 6.8% reduction in mean concentration per year (95% CI: 0.7%, 12.5%). While males tend to have a lower concentration level, the effect is not statistically significant (*p* = 0.193). Graphically, the linear mixed effects model fits reasonably well, although there is some indication of a dip in concentration at 2 h. Full details can be found in Appendix 1.

For 11OHA4, a model accounting for age via assumed pubertal status fits the data slightly better than using a linear effect of age (AIC 609.0 vs. 609.4). In the model using pubertal status, puberty is associated with a 43% reduction (95% CI: 11%–63%) in 11OHA4 levels compared to pre‐puberty.

#### 3.2.3 Estimated Normal Ranges

Based on the above models, the quantiles of concentration level can be calculated at a given time after waking for a given age and sex.

Table 1 gives the estimated median and estimated 95% prediction interval (reference range) for each measure for four distinct example patients (male aged 6, male aged 15, female aged 6, female aged 15). These estimates are given at 30 min, 2 h and 8 h after waking. Note that each of the numbers are point estimates of a percentile of the distribution and given the relatively small sample size in our study there is a high level of uncertainty around each estimate. For example, the upper limit of a male aged 15 years has a bootstrap 95% confidence interval of (193, 347) around the point estimate of 266 pmol/L. Figures showing the median estimate and the corresponding 95% prediction interval, for the full range of times up to 12 h after waking in males and females aged 6 and 16 years can be shown in Appendix 2.

**Table 1:** Estimated values of T, A4, 11KT and 11OHA4 for male and females aged 6 years and 16 years.

	T (pmol/L)	11 KT (pmol/L)	A4 (pmol/L)	11OHA4 (pmol/L)
**Thirty minutes**				
Male, 6 years	34.8 (13.3, 91.4)	27.0 (7.7, 95.2)	143.1 (39.1, 523)	125.2 (19.5, 803)
Male, 15 years	101.3 (38.6, 266)	152.2 (43.2, 536)	137.7 (37.6, 504)	66.7 (10.4, 428)
Female, 6 years	44.2 (16.8, 116)	47.6 (13.5, 167)	194.4 (53.1, 711)	167 (26.1, 1071)
Female, 15 years	44.8 (17.1, 118)	158.8 (45.1, 559)	187.1 (51.1, 685)	89 (13.9, 571)
**Two hours**				
Male, 6 years	28.4 (10.8, 74.5)	23.6 (6.5, 85)	109.9 (29.6, 408)	102.3 (16.0, 656)
Male, 15 years	82.6 (31.5, 217)	132.6 (36.8, 478)	105.8 (28.5, 392)	54.5 (8.5, 350)
Female, 6 years	36.0 (13.7, 94.5)	41.4 (11.5, 149)	149.3 (40.3, 554)	136.3 (21.3, 875)
Female, 15 years	36.5 (13.9, 95.8)	138.4 (38.3, 499)	143.8 (38.8, 533)	72.7 (11.3, 466)
**Eight hours**				
Male, 6 years	26.7 (10.2, 70)	13.6 (3.3, 55.7)	91.9 (22.7, 372)	45.6 (7.1, 292)
Male, 15 years	77.6 (29.6, 204)	76.4 (18.6, 313)	88.4 (21.8, 358)	24.3 (3.8, 156)
Female, 6 years	33.8 (12.9, 88.8)	23.9 (5.8, 97.9)	124.8 (30.8, 505)	60.8 (9.5, 390)
Female, 15 years	34.3 (13.1, 90.1)	79.7 (19.4, 327)	120.1 (29.7, 486)	32.4 (5.1, 208)

## 4 Discussion

In this study we have generated pilot data from healthy children that will inform the development of normative data. It is evident that reference ranges will need to consider age, sex and the time of sampling. Pubertal status is likely to play a role in the interpretation of results, although it seems that age as a linear effect is more suitable to determining reference ranges than presumed pubertal status in these steroid hormones.

Saliva is an ideal medium in which to measure hormones in children, enabling multiple samples to be collected throughout a day including close to the time of waking, with minimal distress and inconvenience. There is evolving evidence that the 11‐oxysteroid pathway metabolites may be superior markers of the quality of CAH treatment and may have some diagnostic utility in common conditions such as exaggerated adrenarche and polycystic ovarian syndrome. However, there is a need to define the normal range of these hormones in saliva before they can be introduced to clinical practice.

Saliva samples were collected by Salivette, and study participants each contributed, on average, six to seven samples. Multiple hormone analyses were performed on single samples with fewer than 10% of samples being inadequate in volume. These data suggest this method of sampling is likely to be acceptable and effective in clinical practice.

There are some matrix problems when using the salivette but only for the more hydrophobic steroids for example, T, A4 and 17OHP. This can cause differences in matrix effect of up to 35%. The 11oxC19 steroids are not affected to any degree.

T was similar between sexes in the younger age group. As expected, there was a significant rise in puberty in males, whilst T concentrations remained similar in females in prepubertal and pubertal age groups. A clear diurnal pattern of T was observed in males, this was less evident in females. Age as a linear effect fitted substantially better than assumed pubertal status.

Serum concentrations of A4 are reported to be higher in females compared to males, particularly in the peri‐pubertal and post pubertal period [13, 14]. We observed that males tend to have lower A4 concentrations than females (*p* = 0.009), supporting previous findings. The salivary T and A4 concentrations reported in this study are consistent with similar data reported recently [8]. There seems to be no effect of age or interaction between sex and age. There also seems to be an initial drop from 30 min from waking to 2 h post waking, followed by a plateau throughout the day.

To our knowledge, this is the first report of the circadian profile of the two 11oxC19 steroids 11KT and 11OHA4. In common with the other hormones reported in this study, concentrations of 11KT and 11OHA4 showed a diurnal profile and reduced in all other time points compared to 30 min after waking (*p* < 0.001). 11KT increased with age for both males and females, with a higher trend for males but this was not statistically significant. 11KT is secreted only from the adrenal gland, and this may account for the smaller sex difference in concentrations of this hormone compared to other androgens derived from the gonads. Previous studies reporting 11KT measurements made in peripheral serum and samples collected during adrenal vein sampling from adult males and females, also reported no gender effect on 11KT [1].

Concentrations of 11OHA4 were often undetectable at later time points, possibly limiting the clinical utility of this salivary biomarker, other than in samples collected shortly after waking. Salivary glands express high levels of HSD11B2 which convert 11β‐hydroxysteroids to their 11‐keto counterparts [15]. This may explain the high number of undetectable salivary concentrations of 11OHA4 (148/325 or 45.5%) in our cohort, particularly later in the day. Although, even when accounting for this, there was a statistically significant reduction in concentration of 11OHA4 (*p* = 0.03) by 7% in mean concentration per year of age. Presumed pubertal status fits slightly better for this steroid hormone only compared to using age as a linear effect. Presumed post pubertal concentrations are approximately 43% lower than in pre‐puberty. The reason for this is unknown.

In all hormones measured in this study a circadian profile was observed, with hormone concentrations being highest 30 min after waking and falling to relatively stable concentrations by 2 h after waking. The true peak is still unknown, but this does highlight that concentration falls throughout the day from 30 min after waking. Further research would be required to ascertain a true peak. Salivary samples can be collected at home 30 min after waking. In contrast, blood samples collected in health care settings are likely to miss the early morning hormone peak. Morning serum androgens have been studied in healthy adult volunteers (*n* = 290), aged 21–95 years [16]. Salivary sampling of 11oxC19 steroids was performed throughout the day in 25 of them, showing a diurnal pattern in males and females in all phases of their menstrual cycle [16]. Our study suggests that this diurnal rhythm is also seen in children. A limitation of this study is that salivary sampling was performed during waking hours only and therefore a full 24‐h profile was not possible. Secretion during sleep is unknown. However, as the participant would need to be woken to obtain a salivary sample, it is unknown as to whether this would also influence concentrations obtained.

For all four hormones reported in this study, we observed an effect of age or sex, and we anticipate that reference ranges will need to take account of these patient characteristics. Pubertal status was not available and is likely to impact hormone concentrations given the associations but reassuringly it seems that age as a linear effect can be utilised to inform reference ranges, particularly compared to presumed pubertal status. If we had used Tanner staging, this difference may be less.

The results indicate that to establish reference ranges for each of the hormones, it is necessary to account for both age and sex. However, the results also indicate that it may be reasonable to assume log‐normal distributions and that the log‐mean concentrations at a given time since waking change linearly with age. We can follow the criterion proposed by Jennen‐Steinmetz and Wellek [17], which is based on ensuring a high probability that the true proportions of patients above and below the reference limits is within some tolerance of the nominal level. When adjusting for age we need to ensure this criterion holds for all ages within the range. For instance, to ensure 80% probabilities that the proportions below and above the lower and upper reference limits lie between 1.5% and 3.5% (for a nominal level of 2.5%), and assuming patients are sampled uniformly across the age range of interest (e.g., 5–16 years), 269 patients would be required per sex. One hundred and fourteen per group gives an 80% chance of being between 1% and 4%. Further details of the sample size calculation are given in Appendix 3. The given sample sizes only depend on the assumption of normality (on the log‐scale) and the appropriateness of linear regression for the analysis. Hence, in principle, they apply regardless of which specific time since waking is of interest and hold for each of the four hormones. However, for 11OHA4, the high number of values below the limit of detection mean that this calculation would only be appropriate for 30‐min post waking, and potentially a higher sample would be needed for later times since a left‐censored regression model would need to be fitted.

It is a limitation of this study, that we have defined prepuberty and puberty/post puberty according to age, and Tanner staging was not performed. The absence of Tanner staging or self‐reported pubertal development may impact the interpretation of hormone concentrations and the generalisability of the results. Tanner staging was omitted as our clinical experience shows that children find this examination uncomfortable, and we wished to minimise any barriers to participation in this healthy volunteer study. Self‐reporting of pubertal development was also not performed, based partly on the discomfort it may cause, but also that it can be inaccurate [18]. Adrenarche was also not recorded, which is likely to impact concentrations of 11oxC19 steroids. Statistical analysis shows that age is related to salivary concentrations of T, A4, 11KT and 11OHA4, rather than presumed pubertal status, therefore reference ranges for age rather than pubertal status can be used. In clinical practice, reference ranges of biochemical measurements that are related to the stage of puberty are often defined by chronological age for ease of use, for example IGF‐I [19]. While this is not ideal, it is common practice. In the case of salivary androgens, it is thought that age can be used for informing reference ranges. Future studies may consider incorporating these assessments for more precise categorisation.

These findings could influence both diagnostic and management strategies for adrenal disorders in children. As 11oxC19 steroids are adrenally derived, compared to other androgens which are also secreted by the gonad, they may be able helpful to determine the source of androgen excess in several conditions, including to guide treatment strategies CAH, PCOS, premature adrenarche and Cushing Syndrome. 11KT is thought to be the dominant bioactive androgen in children during adrenarche and may be responsible for the phenotypic changes seen in both adrenarche and premature adrenarche [20]. 11OHA4 is derived from the adrenal gland, but conversion of 11KT can occur outside the adrenal gland (in adipose tissue) and may be influenced by BMI [16]. For future studies with larger sample sizes, BMI should be considered during statistical analysis.

## 5 Conclusion

We present salivary androgen concentrations from 54 healthy children. These pilot data show that both age and sex affect concentrations of salivary androgens both 30 min after waking and throughout the day. These pilot data have informed a power calculation of the number of participants required to define robust reference data. Further research is required to inform the clinical utility in the diagnosis and monitoring of conditions associated with androgen excess.

## Conflicts of Interest

The authors declare no conflicts of interest.

## Data Availability Statement

The data that support the findings of this study are available from the corresponding author upon reasonable request.

## Appendix 1 Tables showing statistical modelling for testosterone (T), androstenedione (A4), 11 ketotestosterone (11KT) and 11Bhydroxyandrostenedione (11OHA4)

Non‐linear mixed effects model for testosterone (T)

**Table:**

Fixed effect parameters	Estimate	95% CI
$\theta_{1}$ (Terminal mean)	3.528	(3.389, 3.667)
$\theta_{2}$ (Mean decrease)	0.434	(0.087, 0.782)
$\theta_{3}$ (Rate of decrease)	0.969	(−0.034, 1.973)
Age*	0.002	(−0.033, 0.036)
Sex (male)	0.265	(0.096, 0.435)
Age × sex interaction	0.117	(0.071, 0.163)

Random effect parameters	Estimate	
Random intercept SD	0.304	
Residual SD	0.387	

*The age variable is centred around 10.3 years so that $\theta_{1}$ corresponds to the median terminal value for someone aged 10.3 years.

Non‐linear mixed effects model for androstenedione (A4)

**Table:**

Fixed effect parameters	Estimate	95% CI
$\theta_{1}$ (Terminal mean)	4.803	(4.577, 5.029)
$\theta_{2}$ (Mean decrease)	0.602	(0.335, 0.870)
$\theta_{3}$ (Rate of decrease)	0.591	(0.281, 0.901)
Age*	−0.004	(−0.031, 0.023)
Sex (male)	−0.306	(−0.534, −0.079)

Random effect parameters	Estimate	
Random intercept SD	0.611	
Random decline SD	0.505	
Correlation between intercept and decline	−0.468	
Residual SD	0.373	

*The age variable is centred around 10.3 years so that $\theta_{1}$ corresponds to the median terminal value for someone aged 10.3 years.

Linear mixed effects model for 11 ketotestosterone (11KT)

**Table:**

Fixed effect parameters	Estimate	95% CI
Intercept	4.484	(4.270, 4.700)
Time since waking	−0.092	(−0.108, −0.077)
Age*	0.134	(0.078, 0.189)
Sex (male)	−0.315	(−0.597, −0.033)
Age: Sex (male) interaction	0.058	(−0.013, 0.129)

Random effect parameters	Estimate	
Random intercept SD	0.414	
Random slope SD	0.024	
Slope‐intercept correlation	0.470	
Residual SD	0.486	

*The age variable is centred around 10.3 years so that intercept refers to the median log‐concentration of a female aged 10.3 years at time of waking.

Linear mixed effects model for 11Bhydroxyandrostenedione (11OHA4)

**Table:**

Fixed effect parameters	Estimate	95% CI
Intercept	4.885	(4.524, 5.246)
Time since waking	−0.135	(−0.161, −0.108)
Age*	−0.070	(−0.133, −0.007)
Sex (male)	−0.288	(−0.722, 0.146)

Random effect parameters	Estimate	
Residual SD	0.723	
Random intercept SD	0.613	

*The age variable is centred around 10.3 years so that intercept refers to the median log‐concentration of a female aged 10.3 years at time of waking.

## Appendix 2

The following figures give the median estimate and the corresponding 95% prediction interval, for the full range of times up to 12 h after waking in males and females aged 6 and 16 years.

Testosterone

A4

11 keto‐testosterone

11OHA4

## Appendix 3

Sample size calculation

To obtain an appropriate sample size, we adapt the methods considered in Jennen‐Steinmetz and Wellek [17]. They considered a criterion based on ensuring that both the true coverage of the region above the estimated upper reference level and the true coverage of the region below the estimated lower reference level are within some tolerance of the target value, with high probability. In their paper, a homogeneous population is assumed. In the case of age‐adjusted limits estimated by linear regression, we propose to adapt their criterion to ensure that a high probability of adequate tail coverage holds for the full range of ages of interest. Using a similar argument to Bellera and Hanley [21], it is possible to show that if patients are assumed to be sampled uniformly across the age‐range then the worst case occurs at either the minimum or the maximum age where the estimated log‐mean response level $\hat{\mu }$ compared to the true mean μ is distributed

$$\hat{\mu }-\mu ∼N(0,\frac{4\sigma^{2}}{n}).$$

This contrasts to the homogeneous case, where

$$\hat{\mu }-\mu ∼N(0,\frac{\sigma^{2}}{n}).$$

Following the argument in Jennen‐Steinmetz and Wellek [17], the coverage probability in each tail can be expressed in terms of tail probabilities of non‐central t‐distributed random variables. Specifically, adapting eq. (5) in their paper, when a separate linear regression model is fitted for each sex separately, the required condition for the sample size n per sex is:

$$P\left(T\left(n-2,-\frac{\sqrt{n}}{2}z_{q*+\delta }\right)\leq \frac{\sqrt{n}}{2}z_{1-q*}\right)-P\left(T\left(n-2,-\frac{\sqrt{n}}{2}z_{q*-\delta }\right)\leq \frac{\sqrt{n}}{2}z_{1-q*}\right)\geq \beta ,$$

where $q^{*}$ represents the required lower reference level (e.g., 0.025 for a 95% two‐sided reference range), β represents the desired probability of good coverage (e.g., 0.8 for an 80% or higher probability), δ determines the desired degree of accuracy (e.g., 0.01 would imply a desired range ±0.01 around $q^{*}$). Here T(m,ξ) represents a non‐central t‐distributed random variate on m degrees of freedom with non‐centrality parameter ξ, and $z_{q}$ represents the 100×q% point of a standard normal distribution. The equation above can be solved numerically with respect to n with the next integer value above the solution being the recommended sample size.

If instead, we assume that there is a common residual variance across males and females, and hence fit a model with main effects for age and sex and an interaction between age and sex, then again assuming that we sample uniformly across the age range of interest and equal numbers are sampled for males and females, it is possible to show that if a total sample size of n per sex is to be sampled the required condition is:

$$P\left(T\left(2n-4,-\frac{\sqrt{n}}{2}z_{q*+\delta }\right)\leq \frac{\sqrt{n}}{2}z_{1-q*}\right)-P\left(T\left(2n-4,-\frac{\sqrt{n}}{2}z_{q*-\delta }\right)\leq \frac{\sqrt{n}}{2}z_{1-q*}\right)\geq \beta ,$$

which leads to generally smaller sample sizes since the estimation of the variance is shared across both groups. The table below gives the resulting sample size under different desired levels of accuracy. If desired, the approach can be adapted to consider other assumptions about the distribution of ages within the sample and the ratio of males to females.

The table below shows required sample sizes for different reference and tolerance levels assuming β=0.8, under different assumptions about the effect of covariates and the residual variance.

**Table:**

$q^{*}$	δ	*n*		
		Homogeneous^a^	Gender‐specific	Interaction model
0.050	0.0300	44	97	84
0.050	0.0250	64	143	124
0.050	0.0200	101	227	198
0.050	0.0150	181	409	357
0.050	0.0100	409	928	810
0.025	0.0150	70	137	114
0.025	0.0125	102	203	169
0.025	0.0100	161	322	269
0.025	0.0075	288	281	486
0.025	0.0050	652	1319	1104

^a^The values given in this column use the ‘exact’ method in Jennen‐Steinmetz and Wellek [17].
